# Simple models to understand complex disease: 10 years of progress from *Caenorhabditis elegans* models of amyotrophic lateral sclerosis and frontotemporal lobar degeneration

**DOI:** 10.3389/fnins.2023.1300705

**Published:** 2024-01-04

**Authors:** Randall J. Eck, Jade G. Stair, Brian C. Kraemer, Nicole F. Liachko

**Affiliations:** ^1^Graduate Program in Neuroscience, University of Washington, Seattle, WA, United States; ^2^Division of Gerontology and Geriatric Medicine, Department of Medicine, University of Washington, Seattle, WA, United States; ^3^Geriatrics Research Education and Clinical Center, Veterans Affairs Puget Sound Health Care System, Seattle, WA, United States; ^4^Department of Psychiatry and Behavioral Sciences, University of Washington, Seattle, WA, United States; ^5^Department of Laboratory Medicine and Pathology, University of Washington, Seattle, WA, United States

**Keywords:** *C. elegans*, neurodegeneration, amyotrophic lateral sclerosis, frontotemporal lobar degeneration, TDP-43, C9orf72, SOD1, FUS

## Abstract

The nematode *Caenorhabditis elegans* are a powerful model system to study human disease, with numerous experimental advantages including significant genetic and cellular homology to vertebrate animals, a short lifespan, and tractable behavioral, molecular biology and imaging assays. Beginning with the identification of SOD1 as a genetic cause of amyotrophic lateral sclerosis (ALS), *C. elegans* have contributed to a deeper understanding of the mechanistic underpinnings of this devastating neurodegenerative disease. More recently this work has expanded to encompass models of other types of ALS and the related disease frontotemporal lobar degeneration (FTLD-TDP), including those characterized by mutation or accumulation of the proteins TDP-43, C9orf72, FUS, HnRNPA2B1, ALS2, DCTN1, CHCHD10, ELP3, TUBA4A, CAV1, UBQLN2, ATXN3, TIA1, KIF5A, VAPB, GRN, and RAB38. In this review we summarize these models and the progress and insights from the last ten years of using *C. elegans* to study the neurodegenerative diseases ALS and FTLD-TDP.

## Introduction

Amyotrophic lateral sclerosis (ALS) is a devastating neurodegenerative disease characterized by progressive muscle denervation and motor neuron loss in the brain and spinal cord. ALS affects one in 350 individuals, with higher rates of ALS in some populations including military veterans (Tai et al., 2017; van Es et al., 2017). Although the majority of cases of ALS are sporadic, with no known genetic cause, approximately 5%–10% of cases have a familial-inherited causative mutation. To date there are more than 45 human genes implicated as genetic drivers of ALS (Smukowski et al., 2022). ALS-causing gene mutations provide insight into cellular mechanisms that initiate disease and can be a starting point to model ALS in the laboratory. In human disease, most patients with sporadic ALS (sALS) and familial-inherited ALS (fALS) exhibit inclusions of the transactive response DNA binding protein (TDP-43) in disease affected neurons. However, patients with fALS mutations in the *SOD1* gene accumulate aggregates of the protein SOD1, while patients with *FUS* mutations accumulate aggregates of the protein FUS. Approximately half of all patients with frontotemporal lobar degeneration (FTLD), another neurodegenerative disease, also exhibit TDP-43 pathology (FTLD-TDP). A subset of FTLD-TDP patients exhibit motor symptoms, while some ALS patients exhibit FTLD-like cognitive changes. Some genetic causes of ALS can lead to ALS, FTLD-TDP, or mixed ALS/FTLD presentations within the same family, leading to the recognition that ALS and FTLD-TDP represent a clinical spectrum of related diseases (Strong et al., 2017).

The nematode *Caenorhabditis elegans* was established in the 1960s as a tractable model organism for scientific research (Brenner, 1973). *C. elegans* are optically transparent, have a relatively short lifespan averaging 21 days, and can self-fertilize resulting in genetically identical progeny (Johnson, 2003). Adult hermaphrodite *C. elegans* contain ~300 neurons with a defined and consistent connectome controlling sensory, motor, and interneuron signaling with relevant human neurotransmitters such as glutamate, gamma-aminobutyric acid (GABA), acetylcholine, dopamine, and serotonin (Cook et al., 2019). At least 40% of the *C. elegans* protein coding genome, or 7,943 genes, are orthologues or paralogs of human genes, including a significant number of genes related to human genetic disease (Shaye and Greenwald, 2011; Kim et al., 2018). Disease relevant biological pathways are also conserved in *C. elegans* (Shaye and Greenwald, 2011; Kim et al., 2018). These experimental advantages have fueled the use of *C. elegans* to model neurodegenerative diseases (Silverman et al., 2009; Apfeld and Alper, 2018; Caldwell et al., 2020).

**Figure 1:**
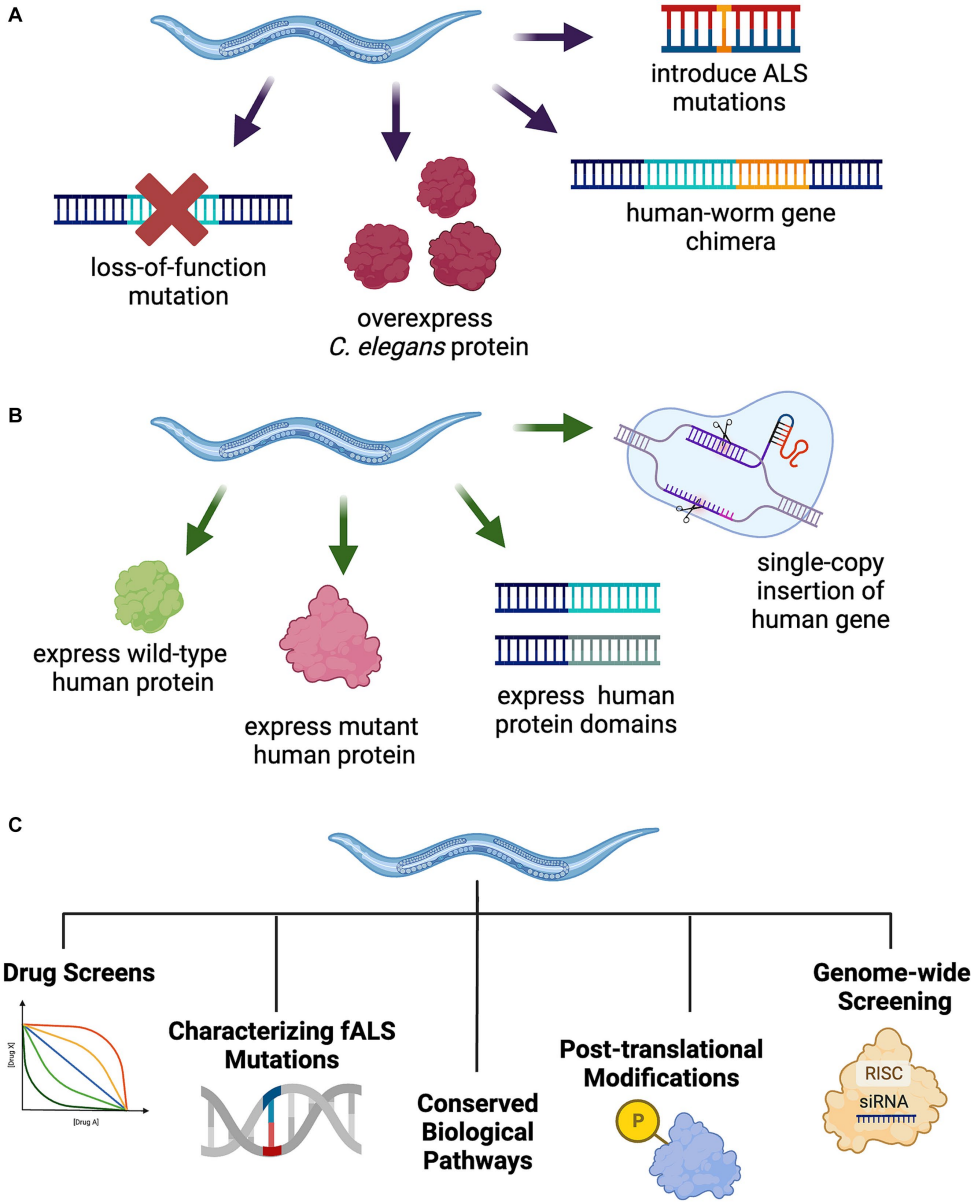
Strategies to model TDP-43-driven ALS and FTLD-TDP in *C. elegans.* **(A)** Manipulation of the endogenous *C. elegans* ALS gene homolog through introduction of fALS mutations at conserved loci, protein overexpression, human-worm gene chimeras, or loss-of-function mutations. **(B)** Generation of transgenic *C. elegans* expressing full-length wild-type or mutant human protein, select protein domains, or a single-copy insertion of the human gene into the genome, potentially replacing the endogenous worm homolog. **(C)** *C. elegans* model utility for a broad range of unbiased and hypothesis driven research including drug screening, fALS mutation characterization, cellular and molecular pathway exploration, protein post-translational modifications, and genome-wide applications including forward and reverse genetics approaches.

Approaches to modeling ALS/FTLD in *C. elegans* either manipulate the endogenous *C. elegans* homolog of a known disease gene or utilize transgenes to express a human disease associated gene (Figure 1). When an ALS/FTLD-associated gene is conserved in *C. elegans*, researchers employ a variety of strategies. These include deletion or partial reduction of the *C. elegans* gene, overexpression, introduction of human ALS/FTLD-associated mutations into the endogenous *C. elegans* homolog at conserved sites, or generation of a chimera of the *C. elegans* protein with key domains from the wild-type or mutant human protein. To directly examine the consequences of human ALS/FTLD genes, researchers can transgenically express wild-type or mutant human disease-associated genes in muscles, neurons, or throughout the *C. elegans* body, express individual protein domains, or replace the *C. elegans* homolog with a single-copy knock-in of the wild-type or mutant human gene. More recent efforts to model neurodegenerative diseases in *C. elegans* have included the development of a photoconvertible fluorescent protein tag to track protein dynamics *in vivo* (Pigazzini and Kirstein, 2020), the conditional expression or inducible aggregation of neurotoxic proteins in aging (Lim et al., 2020), the use of natural genetic variation to study resistance and resilience to protein aggregation in disease (Alexander-Floyd et al., 2020), the study of synergies between distinct pathological proteinopathies (Benbow et al., 2020; Latimer et al., 2022), the exploration of glia–neuron communication in protein quality control (Bar-Ziv et al., 2023), and the development of models to study prion-like seeding or spread of disease-causing proteins in neurons (Gallrein et al., 2021; Zanier et al., 2021). These approaches may inspire future ALS/FTLD models in *C. elegans*.

A comprehensive review of *C. elegans* ALS models was published in 2014 (Therrien and Parker, 2014); however, significant progress has been made in the last 10 years studying genetic and sporadic forms of ALS using *C. elegans*, refining our understanding of ALS and its relationship with FTLD, and identifying new pathways and targets for therapeutic development. This review will focus on these advances from 2014 to 2023, identifying progress and highlighting areas for further investigation.

## *Caenorhabditis elegans* models of ALS/FTLD by proteinopathy

### TDP-43

Cytoplasmic aggregates of TDP-43 are found in the motor neurons of approximately 97% of ALS patients (Arai et al., 2006; Neumann et al., 2006; Tan et al., 2017). Encoded by the *TARDBP* gene, TDP-43 regulates transcription, pre-mRNA and alternative splicing, mRNA stability and transport, and microRNA biogenesis (reviewed in Nilaver and Urbanski, 2023). TDP-43 contains two RNA-binding domains (RRM1 and RRM2), a nuclear localization signal (NLS), and a C-terminal glycine-rich, low-complexity, intrinsically disordered region. More than 30 different mutations have been identified in *TARDBP* that cause fALS (Smukowski et al., 2022). The majority of fALS mutations are located in the C-terminus and potentiate a variety of changes in protein function, including altering TDP-43 liquid–liquid phase separation (reviewed in Hurtle et al., 2023). Both loss of normal TDP-43 functions and gains of toxic function may contribute to disease (as reviewed in Gao et al., 2018; Prasad et al., 2019). For example, loss of TDP-43 nuclear function alters the splicing of thousands of mRNA transcripts, while cytoplasmic aggregates of TDP-43 in disease sequester RNA binding proteins and RNA, which may also contribute to cellular dysfunction (Barmada, 2015; Mehta et al., 2023).

Loss of function mutations in the *C. elegans* TDP-43 homolog *tdp-1* (*tdp-1(ok803) or tdp-1(ok781)*) do not cause motor deficits or neurodegeneration (Table 1) (Ash et al., 2010; Saldi et al., 2018; Mitra et al., 2019). However, loss of *tdp-1* increases *C. elegans* sensitivity to DNA damage and oxidative stress, enhances the efficacy of nuclear RNA interference, produces double stranded RNA foci, and alters exon inclusion in mRNA splicing (Saldi et al., 2014, 2018; Melnick et al., 2019; Mitra et al., 2019; Lins et al., 2023; Taylor et al., 2023). Loss of *tdp-1* also causes temperature dependent lifespan extension (Zhang et al., 2012; Vaccaro et al., 2012c) by modifying DAF-16/FOXO signaling (Table 1) (Zhang et al., 2014). On the other hand, overexpression of TDP-1 in neurons, under the *snb-1* promoter, is sufficient to trigger motor deficits (Ash et al., 2010). TDP-1 overexpression under its endogenous promoter also decreases lifespan of *C. elegans* grown at both 20°C and 25°C (Table 1) (Vaccaro et al., 2012c). Like in human neurons, TDP-1 localizes to cytoplasmic granules during osmotic stress, as visualized by a P*_snb-1_*::TDP-1::YFP fusion (Zhang et al., 2014). A CRISPR-Cas9 generated *tdp-1* true null allele, *tdp-1(tgx58)*, exhibits increased sensitivity to moderate oxidative stress, as evidenced by increased loss of glutamatergic sensory neurons. This phenotype was rescued by insertion of wild-type human TDP-43 sequences at the endogenous *tdp-1* locus, demonstrating a conservation of function (Lins et al., 2023).

**Table 1:** *C. elegans* models of TDP-43-driven ALS and FTLD-TDP.

Pathology	Approach	Transgene	ALS phenotypes	Publication	Follow-up
TDP-43	Deletion	*tdp-1(ok803)*	N/A	Ash et al. (2010)	Mitra et al. (2019), Saldi et al. (2018), Melnick et al. (2019), Zhang et al. (2014), Saldi et al. (2014)
TDP-43	Deletion	*tdp-1(ok781)*	N/A	Zhang et al. (2012)	Saldi et al. (2014)
TDP-43	Overexpression	P*_snb-1_*::TDP-1	Motor deficits	Ash et al. (2010)	
TDP-43	Deletion	*tdp-1(csb38)*	N/A	Taylor et al. (2023)	
TDP-43	Deletion	*tdp-1(tgx58)*	Stress-induced glutamatergic sensory neuron degeneration	Lins et al. (2023)	
TDP-43	Overexpression	P*_tdp-1_*::TDP-1::GFP	Lifespan reduction	Vaccaro et al. (2012c)	
TDP-43	Overexpression	P*_snb-1_*::TDP-1-YFP	N/A	Zhang et al. (2014)	
TDP-43	Wildtype expression	P*_snb-1_*::TDP-43(WT)	Motor deficits, lifespan reduction	Liachko et al. (2010)	Liachko et al. (2016), Jablonski et al. (2015), Taylor et al. (2018), Liachko et al. (2019), Boyd et al. (2014), Currey et al. (2020)
TDP-43	Mutant expression	P*_snb-1_*::TDP-43(A315T)	Motor deficits, lifespan reduction, GABAergic motor neuron degeneration	Liachko et al. (2010)	Liachko et al. (2019), Wong et al. (2018), Boyd et al. (2014), Kow et al. (2022), Currey et al. (2020)
TDP-43	Mutant expression	P*_snb-1_*::TDP-43(M337V)	Motor deficits, lifespan reduction, GABAergic motor neuron degeneration	Liachko et al. (2010)	Liachko et al. (2014), Jablonski et al. (2015), Liachko et al. (2016), Weeks et al. (2018), Liachko et al. (2019), Rojas-Prats et al. (2021), Currey et al. (2020), Lee et al. (2023)
TDP-43	Mutant expression	P*_snb-1_*::TDP-43(G290A)	Motor deficits, lifespan reduction, GABAergic motor neuron degeneration	Liachko et al. (2010)	
TDP-43	Wildtype expression	P*_snb-1_*::TDP-43(WT)	Motor deficits	Ash et al. (2010)	Woo et al. (2017), Koopman et al. (2023a,b,c,d)
TDP-43	Wildtype expression	P*_snb-1_*::TDP-43(WTΔNLS)	N/A	Ash et al. (2010)	
TDP-43	Wildtype expression	P*_snb-1_*::TDP-43(WTΔRRM1)	N/A	Ash et al. (2010)	
TDP-43	Wildtype expression	P*_snb-1_*::TDP-43(WTΔRRM2)	N/A	Ash et al. (2010)	
TDP-43	Wildtype expression	P*_snb-1_*::TDP-43(WTΔc-terminus)	N/A	Ash et al. (2010)	
TDP-43	Wildtype expression	P*_snb-1_*::TDP-43(WTΔcaspases)	Motor deficits	Ash et al. (2010)	
TDP-43	Wildtype expression	P*_snb-1_*::TDP-C25::YFP	Motor deficits	Zhang et al. (2011)	Zhang et al. (2014), Tseng et al. (2023)
TDP-43	Wildtype expression	P*_unc-47_*::TDP-43(WT)	Motor deficits, GABAergic motor neuron degeneration	Vaccaro et al. (2012b)	Veriepe et al. (2015), Aggad et al. (2014), Vaccaro et al. (2012a,c, 2013)
TDP-43	Mutant expression	P*_unc-47_*::TDP-43(A315T)	Motor deficits, GABAergic motor neuron degeneration	Vaccaro et al. (2012b)	Veriepe et al. (2015), Aggad et al. (2014), Bose et al. (2019), Vaccaro et al. (2012a,c, 2013), Tossing et al. (2022), Tauffenberger et al. (2012), Therrien et al. (2013), Labarre et al. (2022)

Transgenic expression of wild-type human TDP-43 or fALS mutant TDP-43(A315T), TDP-43(M337V), and TDP-43(G290A) in *C. elegans* neurons under the *snb-1* promoter results in several ALS associated phenotypes including progressive motor deficits, GABAergic motor neuron degeneration, a reduction in lifespan, disease-associated TDP-43 phosphorylation at epitopes S409/410, and the formation of insoluble TDP-43 aggregates in the nucleus, but not the cytoplasm (Table 1) (Liachko et al., 2010). The *snb-1-*driven expression of human wild-type TDP-43 also results in decreased fecundity and disrupted chemotaxis (Ash et al., 2010; Koopman et al., 2023b,d), in addition to motor deficits (Koopman et al., 2023c). TDP-43 exhibits temperature-sensitive increases in cytoplasmic mislocalization, accompanied by exacerbated aggregation and pathological phosphorylation at S409/S410, highlighting the importance of cytoplasmic mislocalization in disease pathogenesis (Koopman et al., 2023a). In this model, TDP-43’s NLS, both RNA binding domains, and the C-terminus, but not caspase cleavage sites, are required for TDP-43 neurotoxicity (Ash et al., 2010). The TDP-43 C-terminus alone, when expressed in neurons under the *snb-1* promoter, is sufficient to trigger robust aggregation and motor deficits (Zhang et al., 2011). Expressing TDP-43 or TDP-43(A315T) exclusively in GABAergic motor neurons using the *unc-47* promoter also produces progressive motor deficits, axonal GABAergic neuron degeneration, lowered fecundity, reduced chemotaxis, and accumulation of TDP-43 aggregates in both the nucleus and the cytoplasm, but not a reduction in lifespan (Table 1) (Vaccaro et al., 2012b,c). These transgenic overexpression models suggest fALS mutations G290A, A315T, and M337V in the C-terminus of TDP-43 are gain-of-function mutations, since mutant TDP-43 results in more severe motor deficits, neurodegeneration, lifespan reduction, aggregation, and pathological phosphorylation compared to wild-type expression, and these phenotypes do not align with the effects of loss of *tdp-1* function.

TDP-43 phosphorylation serves as a robust and consistent clinical marker of pathological TDP-43 inclusions in ALS patient motor neurons (Arai et al., 2006; Neumann et al., 2006; Hasegawa et al., 2008). In *C. elegans*, the kinases PRKD2/3, CDC7 and TTBK1/2 can phosphorylate TDP-43 *in vivo* (Liachko et al., 2014). *rgef-1* promoter mediated neuronal expression of TTBK1, but not TTBK2, exacerbates motor deficits and increases accumulation and S409/S410 phosphorylation of wild-type TDP-43 (Taylor et al., 2018). These results suggest dysregulation of TDP-43 phosphorylation contributes to the progression of ALS and is a possible therapeutic target (Eck et al., 2021).

In *C. elegans*, there is a long history of using forward genetic and whole genome screens to identify novel gain of function mutations and genes that modify neurodegenerative phenotypes (reviewed in Kutscher and Shaham, 2014, Sin et al., 2014). These screens reveal critical biological pathways and new therapeutic targets in disease. A genome wide RNAi screen identified several modifiers of motor deficits in *C. elegans* expressing TDP-43 and mutant TDP-43(M337V) in neurons. Of these, loss of function mutations in *hse-5(tm472)/GLCE*, *zig-3(tm924)/HMCN1*, *paqr-1(tm3262)/ADIPOR1, gly-8(tm1156)/GALNT11*, and *sax-2(ky216)/FRYL* reduce accumulation and pathological TDP-43 phosphorylation, indicating they act in pathways critical to the development of TDP-43 toxicity (Table 2). In addition, *hse-5* restores synaptic transmission in GABAergic motor neurons (Liachko et al., 2019). Another modifier of mutant TDP-43(M337V) is *rad-23.* RAD-23, and its human homologs RAD23A and RAD23B, are part of the endoplasmic-reticulum (ER) associated protein degradation pathway and function in substrate clearance and DNA damage repair. Loss of function mutations *rad-23(tm3690)* and *rad-23(tm2595)* rescue motor deficits, GABAergic motor neuron degeneration, and TDP-43(M337V) aggregation (Table 2) (Jablonski et al., 2015).

**Table 2:** A list of genes that modify ALS/FTLD-TDP phenotypes in *C. elegans* models of disease.

Pathology	Suppressor	Human homolog	Function	Publication
TDP-43	*hse-5*	*GLCE*	Heparan sulfate modifying enzyme	Liachko et al. (2019)
TDP-43	*zig-3*	*HMCN1*	Secreted immunoglobulin protein	Liachko et al. (2019)
TDP-43	*paqr-1*	*ADIPOR1*	Signaling receptor activity	Liachko et al. (2019)
TDP-43	*gly-8*	*GALNT11*	Polypeptide N-acetylgalactosaminyl transferase	Liachko et al. (2019)
TDP-43	*sax-2*	*FRYL*	Maintaining normal neuronal morphology	Liachko et al. (2019)
TDP-43	*rad-23*	*RAD23*	Proteasomal ubiquitin receptor	Jablonski et al. (2015)
TDP-43	*aly-2*	*ALYREF*	mRNA export	Kow et al. (2022)
TDP-43	*aly-3*	*ALYREF*	mRNA export	Kow et al. (2022)
TDP-43	*tir-1*	*SARM1*	Receptor domain in innate immunity	Veriepe et al. (2015)
TDP-43	*nsy-1*	*MAP3K*	Apoptosis signal-regulating kinase	Veriepe et al. (2015)
TDP-43	*sek-1*	*MAPKK*	Mitogen-activated protein kinase	Veriepe et al. (2015)
TDP-43	*pmk-1*	*p38*	Mitogen-activated protein kinase, innate immunity	Veriepe et al. (2015)
TDP-43	*atf-7*	*ATF7*	Transcription factor in innate immunity	Veriepe et al. (2015)
TDP-43	*unc-13*	*UNC13C*	Calmodulin binding activity and syntaxin-1 binding activity	Veriepe et al. (2015)
TDP-43	*unc-31*	*CADPS2*	Calcium ion binding activity and phosphatidylinositol-4,5-bisphosphate binding activity	Veriepe et al. (2015)
TDP-43	*cnx-1*	*CANX/CLGN*	Type I Ca2 + −binding integral membrane protein of the endoplasmic reticulum	Aggad et al. (2014)
TDP-43	*crt-1*	*CALR*	Calcium-binding molecularchaperone of the endoplasmic reticulum	Aggad et al. (2014)
TDP-43	*unc-68*	*RYR1/2/3*	Ryanodine receptor required for locomotion	Aggad et al. (2014)
TDP-43	*itr-1*	*ITPR1/2/3*	Inositol (1,4,5) trisphosphate receptor	Aggad et al. (2014)
TDP-43	*asp-4*	*CTSD*	Aspartyl protease, required for cell death in neurons	Aggad et al. (2014)
TDP-43	*tra-3*	*CAPN5*	Atypical calpain regulatory protease	Aggad et al. (2014)
TDP-43	*spr-5*	*LSD1*	H3K4me2 demethylase, chromatin remodeling	Periz et al. (2015)
TDP-43	*ufd-2*	*UBE4B*	E4 ubiquitin conjugation factor	Periz et al. (2015)
C9orf72	*daf-2*	*IGF1R*	Receptor tyrosine kinase	Rudich et al. (2017)
C9orf72	*dss-1*	*SEM1*	Subunit of the 26S proteasome regulatory particle	Puleo and Lamitina (2020)
C9orf72	*spop-1*	*SPOP*	Ubiquitin ligase adaptor protein	Snoznik et al. (2021)
C9orf72	*eif-2D*	*eIF2D*	Translation initiation factor activity	Sonobe et al. (2021)
C9orf72	*ulp-3*	*NEDP1*	Involved in protein deneddylation	Kassouf et al. (2023)
C9orf72	*F57A10.2*	*VAPB*	Located in cytoskeleton	Wang et al. (2016)
C9orf72	*acp-4*	*ACP2*	Involved in dephosphorylation	Wang et al. (2016)
SOD1	*spr-5*	*LSD1*	Enables histone H3-di/monomethyl-lysine-4 FAD-dependent demethylase activity	Periz et al. (2015)
SOD1	*ufd-2*	*UBE4B*	Enables chaperone binding activity	Periz et al. (2015)
SOD1	*lin-61*	*L3MBTL1*	Function in a transcriptional regulatory complex	Lu et al. (2019)
SOD1	*kcnl-2*	*SK2*	Small conductance, calcium-activated potassium (K+) channel	Nam et al. (2018)
SOD1	*math-33*	*USP7*	Enables RNA polymerase II-specific DNA-binding transcription factor binding activity	Zhang et al. (2020)
SOD1	*grp-1*	*CYTH4*	Enable guanyl-nucleotide exchange factor activity	Zhai et al. (2015)
SOD1	*efa-6*	*PSD*	Enable guanyl-nucleotide exchange factor activity	Zhai et al. (2015)
SOD1	*rad-23*	*RAD23*	Proteasomal ubiquitin receptor	Jablonski et al. (2015)
SOD1	*figo-1*	*FIG4*	Enable phosphatidylinositol-3,5-bisphosphate 5-phosphatase activity	Osborne et al. (2021)
SOD1	*ulp-3*	*NEDP1*	Involved in protein deneddylation	Kassouf et al. (2023)
FUS	*sqst-1*	*SQSTM1/p62*	Enable K63-linked polyubiquitin modification-dependent protein binding activity	Baskoylu et al. (2022)
FUS	*fsn-1*	*FBXO45*	Enables protease binding activity	Tossing et al. (2022)
FUS	*rpm-1*	*MYCBP2*	E3 ubiquitin ligase	Tossing et al. (2022)
FUS	*klp-7*	*KIF2A/B/C*	Enables kinetochore binding activity	Tossing et al. (2022)
FUS	*dlk-1*	*MAP3K12*	Mitogen-activated protein kinase kinase kinase	Tossing et al. (2022)
FUS	*parp-2*	*PARP2*	A poly(ADP-ribose) polymerase	Tossing et al. (2022)
FUS	*parp-1*	*PARP1*	A poly(ADP-ribose) polymerase	Tossing et al. (2022)
FUS	*tir-1*	*SARM1*	Receptor domain in innate immunity	Veriepe et al. (2015)

In *C. elegans* expressing TDP-43(A315T) in neurons, loss of function mutations in the RNA export factor ALYREF homologs *aly-2(bk3079)* and *aly-3(bk3069)* together rescue motor deficits (Table 2) (Kow et al., 2022). When expressed in GABAergic motor neurons specifically, TDP-43(A315T) triggers an innate immune response in motor neurons and surrounding tissue. Suppressing this innate immune response by a *tir-1(qd4)* deletion allele rescues motor deficits and neurodegeneration without altering TDP-43(A315T) levels. The TIR-1 receptor is critical to the innate immune response and is homologous to human Sarm1, which also regulates axon degeneration. Loss of function mutations in TIR-1 pathway genes *nsy-1(ok593)/MAP3K*, *sek-1(km4)/MAPKK*, *pmk-1(km25)/p38*, and transcription factor *atf-7(qd22)/ATF7* also reduce motor deficits and neurodegeneration. Loss of function mutations in the neurosecretory genes *unc-13(e540)/UNC13C* and *unc-31(e928)/CADPS2* rescue neurodegeneration as well, suggesting neurosecretion is critical to innate immune induction in this model (Table 2) (Veriepe et al., 2015). Another pathway required for TDP-43(A315T) toxicity in GABAergic motor neurons is ER calcium-regulated calpain and aspartyl protease activity. Null mutations *cnx-1(nr2009)/CANX*, *crt-1(bz30)/CALR*, *unc-68*(e540)/*RYR1/2/3*, *itr-1(sa73)/ITPR2/3*, *asp-4(ok2693)/CTSD*, and *tra-3(ok2207)/CAPN5* disrupt this pathway and rescue TDP-43(A315T) driven progressive paralysis as well as reduce neurodegeneration without altering TDP-43(A315T) levels (Table 2). None of these mutants improve *C. elegans* expressing wild-type TDP-43 (Aggad et al., 2014).

Cold temperature, which also extends lifespan, reduces the accumulation of TDP-43 protein in TDP-43(M337V) transgenics grown at 15°C. Knockdown of *psme-3/PSME3*, a proteasome regulator, reverses this reduction (Lee et al., 2023). In addition to aging, protein quality control genes also modify TDP-43 aggregation. Loss of function mutations in *spr-5(by134)/LSD1* and *ufd-2(tm1380)/UBE4B* together dramatically improve motor deficits and reduce the aggregation of C-terminally truncated TDP-43 (TDP-43-C25) by upregulating proteasomal and autophagic degradation (Table 2) (Periz et al., 2015).

Taken together, these modifiers of TDP-43 identified through genetic screens in several *C. elegans* models of ALS represent a group of compelling therapeutic targets and implicate the activity or disruption of ER-associated protein homeostasis, RNA metabolism, protease activity, the innate immune response, proteasomal and autophagic degradation, and other biological pathways in ALS pathogenesis.

In addition to genetic screens, *C. elegans* models enable cost-effective high-throughput screens of drug and novel chemical libraries for basic efficacy and toxicity, shaping the drug discovery pipeline (O’Reilly et al., 2014; Giunti et al., 2021). For ALS, several screens in *C. elegans* models have identified translatable neuroprotective compounds. One screen of 3,768 small molecules in *C. elegans* expressing mutant TDP-43(A315T) in GABAergic motor neurons identified 11 compounds that improve motor deficits when delivered at a concentration of 20 μM for 6 hour in a liquid culture. Of these, the most potent, TRVA242, also decreases neurodegeneration by an unknown mechanism (Bose et al., 2019). *C. elegans* can also screen derivatives of chemical compounds aimed at lowering their necessary dosage for action. *C. elegans* expressing mutant TDP-43(A315T) in neurons pinpointed more effective inhibitors of CDC7 kinase activity, which reduce TDP-43 phosphorylation *in vivo* (Rojas-Prats et al., 2021). In the same model, a screen of ethosuximide-based compounds found α-methyl-α-phenylsuccinimide (MPS) significantly improves motor deficits, rescues shortened lifespan, and reduces GABAergic motor neuron degeneration through a pathway that requires the FOXO transcription factor DAF-16 (Wong et al., 2018).

Outside of large library or derivative screens, *C. elegans* can also validate results from cell-based screens or test known neuroprotective compounds. For example, compound LDN-0130436, identified in a screen of 75,000 compounds in cells, rescues motor deficits and decreases GABAergic motor neuron degeneration in *C. elegans* expressing TDP-43 and mutant TDP-43(A315T) in all neurons (Boyd et al., 2014). Recently, a series of proteolysis targeting chimeras (PROTACs), an alternative to classical chemical agonists, were also screened in a *C. elegans* model expressing the C-terminus of TDP-43 in neurons. PROTACS are designed to scaffold an interaction between a protein of interest and an E3 ligase, which target the protein for degradation by the proteasome. In *C. elegans,* PROTAC2 decreases TDP-43-C25 aggregation and partially rescues motor deficits (Tseng et al., 2023).

Overall, the expression of both wild-type and mutant TDP-43 in *C. elegans* neurons provides a platform for the study of loss and gain of function mechanisms in both sALS and fALS. These models have facilitated the identification of dozens of novel genetic modifiers of TDP-43 proteinopathy in ALS and many neuroprotective chemical compounds.

### C9orf72

The most common genetic cause of ALS is an expansion of the non-coding hexanucleotide repeat G_4_C_2_ in the first intron of the *C9ORF72* gene (DeJesus-Hernandez et al., 2011; Renton et al., 2011). This expansion is relatively rare in Asia and the Middle East; the majority of families affected are of European descent (Majounie et al., 2012). Normally, *C9ORF72*’s first intron contains less than 12 hexanucleotide repeats, but in fALS, this region is oftentimes hundreds or even thousands of hexanucleotide repeats long (Balendra and Isaacs, 2018). There remains disagreement on the threshold of repeat length necessary for fALS, but one meta-analysis suggested repeats as short as 24 may be sufficient (Van Mossevelde et al., 2017; Iacoangeli et al., 2019). These hexanucleotide expansions could contribute to neurodegeneration by altering C9orf72 expression, through the aggregation of repeat RNAs, or through aggregation of dipeptide repeat proteins (DPRs) (Tang et al., 2020). C9orf72 regulates autophagy, endosomal trafficking, lysosomal biogenesis, and inflammation by interacting with Rab-GTPases and other partners (Farg et al., 2014; Sellier et al., 2016; Webster et al., 2016; Burberry et al., 2020; Tang et al., 2020). In human motor neurons and mice, haploinsufficiency of C9orf72 leads to neurodegeneration (Shi et al., 2018; Shao et al., 2019). The G_4_C_2_ expansion can be bidirectionally transcribed into RNA that form foci or hexanucleotide repeat RNA can be translated as five DPRs (poly-GA, poly-GP poly-GR, poly-PA, and poly-PR) following repeat-associated non-AUG-dependent (RAN) translation (Ash et al., 2013; Gendron et al., 2013; Zu et al., 2013; Mori et al., 2013a,b). RNA foci can adopt stable conformations and sequester critical RNA binding proteins, resulting in nucleolar stress and other disruptions in cell culture, potentially contributing to neurodegeneration (Haeusler et al., 2014; Balendra and Isaacs, 2018). On the other hand, DPR expression in zebrafish, mice, *Drosophila*, and cell culture is neurotoxic, especially the expression of poly-GR, poly-PR, and poly-GA, dysregulating translation, phase-separated condensates, RNA binding protein function, and other biological pathways (as reviewed in Balendra and Isaacs, 2018, Tang et al., 2020).

In *C. elegans,* deletion of the *C9ORF72* orthologue, *alfa-1(ok3062)*, accelerates age-related paralysis, increases the rate of GABAergic motor neuron degeneration in aging, and reduces resistance to osmotic stress (Table 3). The motor deficits of *alfa-1* mutants add to the toxicity of mutant TDP-43(A315T) in GABAergic motor neurons, suggesting several pathways may lead to neurodegeneration in ALS (Therrien et al., 2013). More recent work has shown that loss of *alfa-1* also causes defects in lysosomal homeostasis which can be partially rescued by the expression of human C9orf72, suggesting both a pathogenic mechanism and conserved protein function (Corrionero and Horvitz, 2018; Ji et al., 2020). *alfa-1* lacks the G_4_C_2_ repeats responsible for C9orf72 RNA and DPR toxicity.

**Table 3:** *C. elegans* models of C9orf72-driven ALS and FTLD.

Pathology	Approach	Transgene	ALS phenotypes	Publication	Follow-up
C9orf72	Deletion	*alfa-1(ok3062)*	Motor deficits, GABAergic motor neuron degeneration	Therrien et al. (2013)	Ji et al. (2020), Corrionero and Horvitz (2018)
C9orf72	Mutant expression	P*_myo − 3_*::(G_4_C_2_)_5_::GFP	N/A	Lamitina (2022)	
C9orf72	Mutant expression	P*_myo − 3_*::(G_4_C_2_)_20_::GFP	RNA foci	Lamitina (2022)	
C9orf72	Mutant expression	P*_myo − 3_*::(G_4_C_2_)_33_::GFP	RNAi foci, DPR expression	Lamitina (2022)	
C9orf72	Mutant expression	P*_myo − 3_*::(G_4_C_2_)_50_::GFP	RNAi foci, DPR expression	Lamitina (2022)	
C9orf72	Mutant expression	P*_myo − 3_*::(G_4_C_2_)_70_::GFP	RNAi foci, DPR expression	Lamitina (2022)	
C9orf72	Mutant expression	P*_myo − 3_*::(G_4_C_2_)_120_::GFP	RNAi foci, DPR expression	Lamitina (2022)	
C9orf72	Mutant expression	P*_myo-3_*::GR_50_::GFP	Motor deficits	Rudich et al. (2017)	Snoznik et al. (2021), Puleo and Lamitina (2020)
C9orf72	Mutant expression	P*_myo-3_*::PR_50_::GFP	Motor deficits	Rudich et al. (2017)	Snoznik et al. (2021), Puleo and Lamitina (2020)
C9orf72	Mutant expression	P*_myo-3_*::GA_50_::GFP	N/A	Rudich et al. (2017)	
C9orf72	Mutant expression	P*_myo-3_*::PA_50_::GFP	N/A	Rudich et al. (2017)	
C9orf72	Mutant expression	P*_unc-47_*::GR_50_::GFP	Motor deficits	Rudich et al. (2017)	
C9orf72	Mutant expression	P*_unc-47_*::PR_50_::GFP	Motor deficits	Rudich et al. (2017)	
C9orf72	Mutant expression	P*_unc-47_*::GA_50_::GFP	N/A	Rudich et al. (2017)	
C9orf72	Mutant expression	P*_unc-47_*::PA_50_::GFP	N/A	Rudich et al. (2017)	
C9orf72	Mutant expression	P*_snb-1_*::(G_4_C_2_)_75_ΔC9^ubi^	Motor deficits, cholinergic motor neuron degeneration, lifespan reduction	Sonobe et al. (2021)	
C9orf72	Mutant expression	P*_snb-1_*::(G_4_C_2_)_75_	Motor deficits, cholinergic motor neuron degeneration, lifespan reduction	Sonobe et al. (2021)	Kassouf et al. (2023)
C9orf72	Mutant expression	P*_snb-1_*::GA_75_::nLuc	Motor deficits, cholinergic motor neuron degeneration, lifespan reduction	Sonobe et al. (2021)	
C9orf72	Mutant expression	P*_hsp − 16_*::(G_4_C_2_)_29_::GFP	Motor deficits, lifespan reduction	Wang et al. (2016)	
C9orf72	Mutant expression	P*_hsp − 16_*::(G_4_C_2_)_9_::GFP	N/A	Wang et al. (2016)	

The expression of these pure G_4_C_2_ repeats in *C. elegans* muscles under the *myo-*3 promoter is sufficient to trigger the formation of RNA foci and RAN translation of DPRs, even without any accompanying human C9orf72 intronic regions (Table 3). By examining transgenics expressing 5, 20, 33, 50, 70, and 120 G_4_C_2_ repeats, the number of G_4_C_2_ repeats required to initiate RNAi foci formation in *C. elegans* was determined to be greater than 5, but less than 20. For RAN translation of DPRs, the repeat threshold falls between 20 and 33, which aligns with the proposed threshold in humans (Lamitina, 2022).

The RAN translation of G_4_C_2_ repeats can result in five DPR proteins depending on the open-reading frame, including (PA)_n_, (GA)_n_, (PR)_n_, and (GR)_n_. In *C. elegans*, the individual expression of DPRs (GR)_50_ and (PR)_50_, but not (PA)_50_ and (GA)_50_, in muscles under the *myo-*3 promoter significantly accelerates age-related paralysis (Table 3) (Rudich et al., 2017; Puleo and Lamitina, 2020; Snoznik et al., 2021). (PR)_50_ DPRs are soluble and localize to the nucleolus. At least 25 repeats and continued expression of poly-PR DPRs are required for paralysis. The forced nuclear localization of (PR)_50_ DPRs accelerates paralysis, while mutations decreasing the rate of physiological aging delay DPR toxicity (Rudich et al., 2017).

A pilot and then genome-wide RNAi screen in *C. elegans* expressing (PR)_50_ DPRs in muscles identified several novel modifiers of C9orf72 toxicity. Two were further characterized: *dss-1* and *spop-1* (Table 2) (Puleo and Lamitina, 2020; Snoznik et al., 2021). Knockdown of *dss-1*, whose human homolog *Sem1* functions in mRNA export and nuclear pore function, partially rescues a progressive paralysis phenotype in both (PR)_50_ and (GR)_50_ expressing transgenic animals but does not alter poly-PR nuclear localization (Puleo and Lamitina, 2020). Loss of function mutations in *spop-1*, whose human homolog SPOP is a nuclear ubiquitin ligase adaptor protein, similarly rescues a progressive paralysis phenotype in (PR)_50_ and (GR)_50_ models without changing DPR levels or localization. The increased activity of transcription factor BET-1, BRD2/3/4 in humans, which is normally degraded by SPOP, is responsible for *spop-1* suppression (Snoznik et al., 2021).

The expression of pure DPRs as well as G_4_C_2_ repeats in cell types other than muscles also results in pronounced motor deficits in *C. elegans*. For example, the expression of DPRs (GR)_50_ and (PR)_50_, but not (PA)_50_ and (GA)_50_, in GABAergic motor neurons under the *unc-47* promoter results in motor deficits and GABAergic motor neuron degeneration (Rudich et al., 2017). In addition, the pan-neuronal expression of 75 G_4_C_2_ repeats flanked by the human C9orf72 intronic regions under the *snb-1* promoter results in the expression of several DPRs, progressive motor deficits, cholinergic motor neuron degeneration, and a reduced lifespan. Lifespan is also reduced when this construct is expressed under a fragment of the *unc-11* promoter, known to be more exclusively active in neurons. The inclusion of the C9orf72 intronic regions allows for the robust translation of poly-GA, which generally requires an initiation codon. The neurodegenerative phenotypes of transgenics lacking poly-GA are less severe (Table 3) (Sonobe et al., 2021).

In this model, loss of function mutations in *C. elegans eif-2D,* or deletion of its SUI1 initiation codon recognition domain, dramatically reduce the expression of poly-GA, suggesting *eif-2D* human homolog eIF2D is critical for DPR translation (Table 2) (Sonobe et al., 2021). Loss of *ulp-3(tm1287),* whose human homolog NEDP1 regulates stress granule dynamics, also rescues motor deficits in this model and reduces the number of stress granule formed during oxidative stress (Table 2) (Kassouf et al., 2023).

The expression of both nine and 29 G_4_C_2_ repeats under the global *hsp-16* promoter results in motor deficits and a reduction in lifespan as well, with 29 G_4_C_2_ repeats resulting in significantly worse phenotypes, suggesting neurotoxicity is repeat length-dependent (Table 3) (Wang et al., 2016). In this model, loss of function mutations in *F57A10.2/VAPB* and *acp-4(gk833833)/ACP2* rescue progressive motor deficits (Table 2). Initially identified in a forward-genetic mutagenesis screen, F57A10.2 is a homolog of human VAPB, which regulates endosomal trafficking, and ACP-4 is the homolog of ACP2, a lysosomal acid phosphatase (Wang et al., 2016).

Taken together, the expression of G_4_C_2_ repeats in *C. elegans* is a valuable model for studying C9orf72 fALS. The expression of C9orf72 DPRs in *C. elegans* produce a variety of neurodegenerative phenotypes that have allowed for the identification of several novel modifiers of DPR toxicity. These modifiers suggest autophagy, the ubiquitin proteasome system, RAN translation, and stress granules as possible therapeutic targets in C9orf72 fALS.

### SOD1

Mutations in the gene Cu/Zn superoxide dismutase 1 (*SOD1*) were the first identified genetic cause of fALS and account for approximately 8–23% of familial and 1–4% of sporadic cases without a family history of ALS (Rosen et al., 1993; Muller et al., 2022; Smukowski et al., 2022). To date more than 150 mutations throughout the SOD1 coding region have been identified, although the most commonly observed in ALS are SOD1 D90A, A4V, or G93A (Pansarasa et al., 2018). For these individuals, neuronal inclusions of the protein SOD1 are the major neuropathological marker. SOD1 localizes to mitochondria and protects these organelles from oxidative stress damage by converting harmful superoxide anions into hydrogen peroxide and oxygen. SOD1 has four cysteine residues that contribute to its stability and catalytic activity, and changes in the redox states of these residues have been found in ALS-causing mutant SOD1 (Peggion et al., 2022). Numerous mechanisms have been proposed by which changes in SOD1 function cause or contribute to ALS, including altered SOD1 protein maturation or localization, increased cellular oxidative stress, or impaired mitochondrial function.

Human SOD1 and *C. elegans* SOD-1 are highly homologous, sharing 71% protein similarity (Baskoylu et al., 2018). In addition to SOD1, there are two other SOD isoforms: MnSOD (manganese superoxide dismutase) and ECSOD (extracellular superoxide dismutase). MnSOD is encoded by the gene SOD2 and ECSOD is encoded by the gene SOD3 (Miao and St. Clair, 2009). However, there are five superoxide dismutase (*sod*) genes in *C. elegans: sod-1* and *sod-5* encode Cu/Zn SOD, *sod-2* and *sod-3* encode MnSOD, and *sod-4* encodes ECSOD. These *sod* genes all have some degree of overlapping or compensatory functions, and coordinated gene expression regulation. For example, *sod-5* expression is increased in a *sod-1* mutant background (Yanase et al., 2009), while *sod-1, sod-2, sod-3,* and *sod-4* expression are all increased in a *sod-5* deletion mutant background (Table 4) (Yanase et al., 2020). In contrast, expression of *sod-4* and *sod-5* are decreased in a *sod-2(gk257);sod-3(tm760)* loss of function background (Back et al., 2010). An earlier study found no effect of *sod* loss of function mutations *sod-1(tm776), sod-2(gk257), sod-3(tm760), sod-4(gk101),* or *sod-5(tm1146)* on lifespan, although they had differential effects on development, fertility, and oxidative stress resistance (Doonan et al., 2008). A subsequent study found *sod-1(tm776)* or *sod-5(tm1146)* decrease lifespan compared to wild-type controls, and the double mutant *sod-1(tm776); sod-5(tm1146)* has a further shortened lifespan. Both insulin/ insulin-like growth factor and p38 MAPK signaling pathways coordinate *sod* gene regulation (Yanase et al., 2009, 2020). Under conditions of oxidative stress, loss of function *sod-1(tm776)* animals exhibit loss of glutamatergic but not cholinergic neurons (Table 4) (Baskoylu et al., 2018).

**Table 4:** *C. elegans* models of SOD1-driven ALS.

Pathology	Approach	Transgene	ALS phenotypes	Publication	Follow-up
SOD-1	Deletion	*sod-1(tm776)*	Stress induced glutamatergic neurodegeneration	Doonan et al. (2008)	Baskoylu et al. (2018), Back et al. (2010), Yanase et al. (2009, 2020), Horspool and Chang (2017), Yu and Chang (2022)
SOD-1	Mutant expression	P*_unc-25_*::SOD1(G93A)::GFP	Motor deficits, age-dependent paralysis, motor neuron degeneration	Li et al. (2013)	
SOD-1	Wildtype expression	P*_unc-25_*::SOD1(WT)::GFP	Motor deficits, age-dependent paralysis, motor neuron degeneration	Li et al. (2014)	
SOD-1	Mutant expression	P*_unc-25_*::SOD1(G93A)::GFP	Motor deficits, age-dependent paralysis, motor neuron degeneration	Li et al. (2014)	
SOD-1	Mutant expression	P*_unc-25_*::SOD1(G93A)::GFP	Motor deficits, motor neuron degeneration, reduced lifespan	Xu et al. (2022)	
SOD-1	Wildtype expression	P*_snb-1_*::SOD1(WT)::YFP	Motor deficits	Wang et al. (2009)	Jablonski et al. (2015), Baskoylu et al. (2018), Lim et al. (2012), Zhai et al. (2015), Boccitto et al. (2012), Zhang et al. (2012, 2020), Nam et al. (2018)
SOD-1	Mutant expression	P*_snb-1_*::SOD1(G85R)::YFP	Motor deficits, synaptic transmission defects	Wang et al. (2009)	Jablonski et al. (2015), Baskoylu et al. (2018), Lim et al. (2012), Zhai et al. (2015), Boccitto et al. (2012), Zhang et al. (2012, 2020), Nam et al. (2018), Tsioras et al. (2023)
SOD-1	Knock-in mutant	P*_sod-1_*::SOD-1(A4V)	Stress-induced cholinergic motor neuron degeneration, reduced lifespan	Baskoylu et al. (2018)	
SOD-1	Knock-in mutant	P*_sod-1_*::SOD-1(H71Y)	Stress-induced cholinergic motor neuron degeneration, reduced lifespan	Baskoylu et al. (2018)	
SOD-1	Knock-in mutant	P*_sod-1_*::SOD-1(L84V)	Reduced lifespan	Baskoylu et al. (2018)	
SOD-1	Knock-in mutant	P*_sod-1_*::SOD-1(G85R)	Stress-induced cholinergic motor neuron degeneration, reduced lifespan	Baskoylu et al. (2018)	Osborne et al. (2021), Kassouf et al. (2023)
SOD-1	Knock-in mutant	P*_sod-1_*::SOD-1(G93A)	Stress-induced cholinergic motor neuron degeneration, reduced lifespan	Baskoylu et al. (2018)	Tossing et al. (2022)
SOD-1	Knock-in mutant	P*_sod-1_*::SOD-1(G85R)	Motor deficits, heightened pathogen avoidance response	Yu and Chang (2022)	
SOD-1	Knock-in mutant	P*_sod-1_*::SOD-1(G93A)	Motor deficits	Yu and Chang (2022)	
SOD-1	Mutant expression	P*_snb-1_*::SOD1(L42Q)::YFP	N/A	Zhong et al. (2017)	
SOD-1	Mutant expression	P*_snb-1_*::SOD1(G85R)::YFP	Motor deficits, reduced lifespan	Zhong et al. (2017)	
SOD-1	Mutant expression	P*_snb-1_*::SOD1(G85R/L42Q)::YFP	Motor deficits, reduced lifespan	Zhong et al. (2017)	
SOD-1	Wildtype expression	P*_unc-14_*::SOD1(WT)::EGFP	Motor deficits	Ogawa et al. (2015)	
SOD-1	Mutant expression	P*_unc-14_*::SOD1(G85R)::EGFP	Motor deficits	Ogawa et al. (2015)	
SOD-1	Mutant expression	P*_unc-14_*::SOD1(C^4^S)::EGFP	N/A	Ogawa et al. (2015)	

To create a model of human SOD1-driven ALS in *C. elegans*, fALS mutant SOD1(G85R) tagged with YFP was expressed pan-neuronally under the *snb-1* promoter. This model induces motor deficits and motor neuron dysfunction, which are exacerbated by knockdown of protein turnover, chaperone, and protein modification genes (Table 4) (Wang et al., 2009). Another pan-neuronal SOD1 model expresses wild-type or mutant SOD1(G93A) tagged with GFP in GABAergic motor neurons under the *unc-25* promoter (Li et al., 2013, 2014). These models exhibit several fALS phenotypes including SOD1 aggregation, motor deficits, age-dependent paralysis, axon guidance defects, and motor neuron degeneration, but no reduction in lifespan. Mutant SOD1(G93A) forms larger and more insoluble aggregates in the axons of motor neurons, correlating with more severe neurodegeneration compared to wild-type SOD1 (Table 4) (Li et al., 2014). Another model expressing SOD1(G93A) under the *unc-25* promoter did have a reduced lifespan in addition to motor deficits and neurodegeneration (Table 4) (Xu et al., 2022).

*C. elegans* models overexpressing human mutant SOD1 preclude the evaluation of loss of function mechanisms of fALS variants. To overcome this, a series of human fALS SOD1 mutations, A4V, H71Y, L84V, G85R, or G93A, were introduced into conserved sites in the endogenous *C. elegans sod-1* gene (Baskoylu et al., 2018). These strains exhibit glutamatergic and cholinergic neuron degeneration when exposed to oxidative stress, while GABAergic, dopaminergic and serotonergic neurons are relatively spared. When crossed with transgenic *C. elegans* expressing human wild-type SOD1::YFP in motor neurons, *sod-1* A4V, H71Y, G85R, or G93A, but not L84V, increase inclusion formation of human SOD1::YFP. Loss of function *sod-1(tm776)* has no effect on SOD1::YFP aggregation, suggesting these mutations have a gain of function mechanism. *sod-1* mutants H71Y, L84V, and G85R show similar stress-induced glutamatergic neuron dysfunction as *sod-1* loss of function, indicating these mutations may have a loss of function component (Table 4) (Baskoylu et al., 2018). Additional single-copy knock-in models of SOD-1(G85R) and SOD-1(G93A) support G85R but not G93A as a loss of function fALS mutation (Yu and Chang, 2022). *C. elegans* lacking *sod-1(tm776)* or expressing SOD-1(G85R), but not SOD-1(G93A), exhibit a heightened pathogen avoidance response to the bacteria *P. aeruginosa*, by regulating the synaptic density of AMPA-type glutamate receptor GLR-1 in the ventral nerve cord (Table 4) (Horspool and Chang, 2017; Yu and Chang, 2022).

In humans, SOD1 shuttles between the nucleus and cytoplasm. In *C. elegans*, the fALS SOD1(G85R) or SOD1(L42Q/G85R) disrupt SOD1 nuclear localization. Expression of these mutations in neurons under the *snb-1* promoter results in cytoplasmic SOD1 aggregates, motor deficits, and a reduced lifespan compared to wild-type SOD1 or SOD1(L42Q) (Table 4) (Zhong et al., 2017), supporting a role for SOD1 mislocalization in disease pathogenesis. In addition, SOD1’s cysteine residues are also necessary for SOD1 toxicity. The expression of human EGFP tagged SOD1 with four cystine residues mutated to serines (SOD1(C^4^S)) in neurons under the *unc-14* promoter results in no motor deficits compared to wild-type *C. elegans*. This is in contrast to the expression of wild-type SOD1 or mutant SOD1(G85R) which display significant motor deficits (Table 4) (Ogawa et al., 2015), indicating the thiol/thiolate state of SOD1’s cystine residues modify fALS SOD1 neurotoxicity.

Forward genetic mutagenesis screens are a powerful method to identify novel mutations that modify a phenotype of interest. A mutagenesis screen in *C. elegans* expressing SOD1(G85R) under the pan-neuronal promoter *snb-1* identified a combination of loss of function mutations in *spr-5/LSD1* and *ufd-2/UBE4B* that suppress SOD1-induced neurotoxicity*. spr-5(by134);ufd-2(tm1380)* improves motor deficits and reduces SOD1(G85R) aggregation by upregulating proteasomal and autophagic degradation, partially through the FOXO transcription factor DAF-16 (Table 2) (Periz et al., 2015). A similar screen in the same model found mutations in *lin-61*, whose human homolog L3MBTL1 regulates p53 protein quality control pathways, rescues SOD1(G85R) motor deficits and aggregation (Table 2) (Lu et al., 2019). A single gain of function mutation in the *C. elegans* gene *kcnl-2(rt462)*, which confers calcium sensitivity in an SK2 channel responsible for neuron excitability, also rescues motor deficits in mutant SOD1(G85R), but not wild-type SOD1, models (Nam et al., 2018). In addition to mutagenesis, a genome-wide RNAi screen identified knockdown of *math-33* rescues motor deficits and SOD1(G85R)::YFP aggregation. The human homolog of MATH-33 is USP7, a ubiquitin protease (Table 2) (Zhang et al., 2020).

The disruption of proteins such as cytohesins and ADP-ribosylation factor (ARF) GTPases have also been implicated in SOD1-linked ALS. In the same model of mutant SOD1(G85R) neuron aggregation, knockdown of *C. elegans* cytohesin homologs *grp-1* and *efa-6* rescues motor defects (Table 2). Loss of function mutations in *C. elegans arf-6(tm1447)* and *arf-1.2(ok796)* do not alter SOD1(G85R) motor deficits, indicating cytohesins but not ARF GTPases are possible therapeutic targets critical for SOD1 neurotoxicity (Zhai et al., 2015). Another modifier of mutant SOD1(G85R) in this model is *rad-23.* RAD-23 and its human homolog are part of the endoplasmic-reticulum (ER) associated protein degradation pathway and function in substrate clearance and DNA damage repair. Loss of function mutations *rad-23(tm3690)* and *rad-23(tm2595)* rescue motor deficits and increase SOD1(G85R) solubility (Table 2) (Jablonski et al., 2015). More recently, a deletion allele of the *C. elegans* homolog of VCP, *cdc-48.1(tm544)*, a fALS gene that regulates protein quality control and intracellular signaling, was found to exacerbate SOD1(G85R)-dependent motor deficits. Overexpression of *cdc-48.1* under its endogenous promoter significantly rescues these motor deficits and SOD1(G85R) aggregation in *C. elegans* neurons, linking two fALS genes and further implicating disruptions to protein quality control in ALS (Tsioras et al., 2023).

To determine if fALS SOD1 mutations interact with other ALS-risk factor genes or fALS genes in disease, the *C. elegans* homologs of ALS/FTLD related genes *figo-1(tm5202)/Fig 4, sqst-1(ok2892)/SQSTM1, ubql-1(tm1574)/UBQLN2, ptl-1(tm543)/MAPT,* or *daao-1(tm3673)/DAO,* were deleted in a single-copy knock-in model of SOD-1(G85R) and wild-type control. Of these, only loss of *figo-1(tm5202)* reduces glutamatergic neuron degeneration by SOD-1(G85R). Therefore, loss of *figo-1* partially compensates for the loss of *sod-1* function, possibly through its indirect function in endosomal signaling and trafficking (Table 2) (Osborne et al., 2021). The expression SOD-1(G85R) also disrupts another conserved ALS-related pathway: stress granules. During oxidative stress, *C. elegans* with the SOD-1(G85R) mutation form larger stress granules as visualized by the G3BP1 homolog *g3bp-1::GFP*, which constitute the stress granule core. Loss of *ulp-3(tm1287)*, whose human homolog NEDP1 regulates stress granule dynamics, reduces the formation of stress granules and rescues SOD-1(G85R) motor deficits, indicating stress granule formation may facilitate ALS-related neuron dysfunction (Table 2) (Kassouf et al., 2023).

Neuroprotective compounds have also been identified in SOD1 fALS models. For example, the diabetes drug metformin rescues motor deficits, reduces neurodegeneration, and extends lifespan in *C. elegans* expressing mutant SOD1(G93A) in GABAergic motor neurons. The beneficial effect of metformin is eliminated when *C. elegans* genes *daf-16* and *lgg-1* are knocked down, indicating metformin likely improves ALS phenotypes by upregulating autophagy (Xu et al., 2022).

Modeling ALS-linked SOD1 in *C. elegans* has provided valuable insights as to its function in disease as well as potential therapeutic methods. Future studies will continue to reveal mechanisms underlying SOD1’s toxicity and pathogenesis.

### FUS

The DNA/RNA binding protein fused in sarcoma (*FUS*) has been identified as another ALS causative gene, with *FUS* mutations accounting for 1%–2% of sporadic ALS cases and 1%–5% of fALS. fALS FUS mutations are predominantly in the C-terminal low complexity, RGG-binding, or nuclear localization signal (NLS) domains. These mutations cause FUS mislocalization to the cytoplasm and aggregation, and are frequently associated with a younger onset of disease (Ishigaki and Sobue, 2018; Birsa et al., 2020). FUS functions as a DNA and RNA binding protein, and regulates DNA damage repair, mRNA splicing and transport, and stress granule formation. Evidence from disease models supports both FUS toxic loss- and gain-of-function mechanisms in disease, potentially through impaired DNA damage repair or RNA splicing defects (Shang and Huang, 2016).

The ortholog for FUS in *C. elegans* is FUST-1. Deletion of *fust-1(tm4439)* recapitulates several fALS phenotypes including progressive motor deficits and axon degeneration, as well as disrupts miRNA-mediated gene silencing and alters exon inclusion in mRNA splicing, suggesting a possible pathogenic loss of function mechanism (Table 5) (Therrien et al., 2016; Zhang et al., 2018; Taylor et al., 2023). In cholinergic motor neurons, FUST-1 localizes to the nucleus, as visualized by an N-terminal GFP tagged construct expressed under the *unc-17* promoter. fALS mutations FUS(R524S) and FUS(P525L) may disrupt the nuclear localization FUS. When these fALS mutations are introduced at conserved sites in the *C. elegans* gene and expressed in cholinergic motor neurons under the *unc-17* promoter with a fluorescent tag, GFP::FUST-1(R446S) and GFP::FUST-1(P447L), but not wild-type GFP::FUST-1, mislocalize to the cytoplasm, where they form aggregates following ER-stress induction (Baskoylu et al., 2022). These *fust-1* mutants exhibit decreased survival when exposed to ER or oxidative stress, impaired autophagy, and neuromuscular junction (NMJ) dysfunction, but not lifespan reduction or neurodegeneration (Table 5). Human SQSTM1/p62 selects cargo for autophagic degradation and accumulates in FUS fALS patient neurons. A loss of function mutation in the *C. elegans* SQSTM1 homolog *sqst-1(ok2892)* rescues or partially rescues *fust-1* mutation driven NMJ and motor deficits (Table 2) (Baskoylu et al., 2022).

**Table 5:** *C. elegans* models of FUS-driven ALS.

Pathology	Approach	Transgene	ALS phenotypes	Publication	Follow-up
FUS	Deletion	*fust-1(tm4439)*	Motor deficits, GABAergic motor neuron degeneration	Therrien et al. (2016)	Zhang et al. (2012, 2018)
FUS	Deletion	*fust-1(csb21)*	N/A	Taylor et al. (2023)	
FUS	Overexpression	P*_unc-17_::*GFP::*fust-1*(WT)	N/A	Baskoylu et al. (2022)	
FUS	Mutant expression	P*_unc-17_::fust-1*(R524S)	Stress-induced lifespan reduction, stress-induced motor deficits, neuromuscular junction impairment, neuronal autophagy impairment	Baskoylu et al. (2022)	
FUS	Mutant expression	P*_unc-17_*::*fust-1*(P525L)	Stress-induced lifespan reduction, stress-induced motor deficits, neuromuscular junction impairment, neuronal autophagy impairment	Baskoylu et al. (2022)	
FUS	Wildtype expression	P*_rgef-1_*::FUS(WT)	Motor deficits	Murakami et al. (2012)	Markert et al. (2020), Murakami et al. (2015)
FUS	Mutant expression	P*_rgef-1_*::FUS(G501Δ)	Motor deficits, reduced lifespan	Murakami et al. (2012)	Markert et al. (2020), Murakami et al. (2015)
FUS	Mutant expression	P*_rgef-1_*::FUS(S513Δ)	Motor deficits, reduced lifespan	Murakami et al. (2012)	
FUS	Mutant expression	P*_rgef-1_*::FUS(R514G)	Motor deficits	Murakami et al. (2012)	
FUS	Mutant expression	P*_rgef-1_*::FUS(R521G)	Motor deficits	Murakami et al. (2012)	
FUS	Mutant expression	P*_rgef-1_*::FUS(R522G)	Motor deficits, reduced lifespan	Murakami et al. (2012)	Murakami et al. (2015)
FUS	Mutant expression	P*_rgef-1_*::FUS(R524S)	Motor deficits	Murakami et al. (2012)	Murakami et al. (2015)
FUS	Mutant expression	P*_rgef-1_*::FUS(P525L)	Motor deficits, reduced lifespan	Murakami et al. (2012)	Murakami et al. (2015), Lee et al. (2023)
FUS	Mutant expression	P*_rgef-1_*::FUS(R495X)	Motor deficits	Murakami et al. (2012)	Murakami et al. (2015)
FUS	Wildtype expression	P*_rgef-1_*::FUSLC(WT)	Motor deficits, increased urea-soluble FUS assemblies, reduced lifespan	Murakami et al. (2015)	
FUS	Mutant expression	P*_rgef-1_*::FUSLC(S96Δ)	Motor deficits, increased urea-soluble FUS assemblies, reduced lifespan	Murakami et al. (2015)	
FUS	Wildtype expression	P*_unc-47_*::FUS(WT)	Motor deficits, age-dependent paralysis, GABAergic motor neuron degeneration	Vaccaro et al. (2012b)	Vaccaro et al. (2012c), Veriepe et al. (2015), Labarre et al. (2022)
FUS	Mutant expression	P*_unc-47_*::FUS(S57Δ)	Motor deficits, age-dependent paralysis, GABAergic motor neuron degeneration	Vaccaro et al. (2012b)	Vaccaro et al. (2012a,c), Tauffenberger et al. (2012), Veriepe et al. (2015)
FUS	Single-copy wildtype	P*_unc-47_*::FUS(WT)	N/A	Labarre et al. (2021)	
FUS	single-copy mutant	P*_unc-47_*::FUS(S57Δ)	Age-dependent paralysis, GABAergic motor neuron degeneration	Labarre et al. (2021)	

The expression of mutant human FUS(R521G, R522G, P525L, and C-terminal truncated FUS513 and FUS501), but not wild-type or FUS(R514G) and FUS(R521G), under the neuron-specific *rgef-1* promoter also causes several fALS phenotypes including motor deficits, a reduced lifespan, cytoplasmic FUS mislocalization, and FUS aggregation (Murakami et al., 2012). In contrast, the expression of human wild-type FUS, mutant FUS(P525L), or FUS(FUS501) without the FUS N-terminal low complexity (LC) domain show reduced FUS aggregation, motor deficits, and lifespan defects. *rgef-1-*driven neuronal expression of the FUS LC domain alone or mutant FUS(S96del) is sufficient to increase FUS aggregation compared to wild-type FUS. Mutant FUS aggregates sequester ribonucleoprotein granule components, preventing new protein synthesis in axon terminals (Table 5) (Murakami et al., 2015). Compared to wild-type FUS, the cholinergic and GABAergic NMJs of *C. elegans* expressing neuronal truncated FUS501 are enriched for endosome-like organelles correlating with increased synaptic and vesicle dysfunction, highlighting another potential gain of function mechanism of mutant FUS (Markert et al., 2020). In the pan-neuronal mutant FUS(P525L) model, cooler growth temperatures, incubating *C. elegans* at 15°C rather than 20°C or 25°C, reduce the accumulation of FUS aggregates. Knockdown of *psme-3/PSME3*, a proteasome regulator, reverses this reduction (Table 2) (Lee et al., 2023).

Expression of human wild-type or mutant FUS(S57Δ) exclusively in GABAergic motor neurons under the *unc-47* promoter also induces motor deficits, neurodegeneration, and the formation of FUS aggregates (Table 5) (Vaccaro et al., 2012b). Overexpression of mutant FUS(S57Δ) in GABAergic motor neurons triggers an innate immune response in these neurons and surrounding tissue. Deletion of innate immunity receptor TIR-1, by *tir-1(qd4)*, rescues motor deficits and neurodegeneration without altering FUS levels, suggesting the innate immune response contributions to FUS neurotoxicity (Table 2) (Veriepe et al., 2015). The expression of a single-copy mutant FUS(S57Δ) under the *unc-47* promoter is sufficient to induce age-dependent motor deficits and neurodegeneration. A single-copy wild-type FUS does not exhibit fALS-associated phenotypes (Table 5) (Labarre et al., 2021). The axonal degeneration and motor deficits in this single-copy FUS(S57Δ) model are dependent on several genes in the DLK-1/MAP3K12 axonal regeneration pathway (Tossing et al., 2022). Knockdown of *fsn-1/FBXO45, rpm-1/MYCBP2, klp-7/KIF2C,* or *dlk-1/MAP3K12* improve a progressive paralysis phenotype, while knockdown of *fsn-1/FBXO45, rpm-1/MYCBP2*, or *parp-2/PARP2* decrease axon degeneration. A loss of function mutation in *parp-1*(ok988)*/PARP1* similarly rescues paralysis and degeneration (Table 2). In fact, a screen of PARP inhibitors identified several compounds that reduce FUS-driven axon degeneration. The PARP inhibitors Olaparib, Veliparib, and 3-AB also partially reduce axon degeneration in mutant SOD1(G93A) and TDP-43(A315T) models (Tossing et al., 2022). In addition to screening drugs, *C. elegans* can also model dietary interventions for ALS. For example, fatty acids derived from the bacteria *Lacticaseibacillus rhamnosus* HA-114 reduce aberrant lipid accumulation, motor deficits, and neurodegeneration in *C. elegans* expressing mutant FUS(S57Δ) or TDP-43(A315T) in GABAergic motor neurons. This rescue acts through genes involved in lipid homeostasis and mitochondrial β-oxidation, suggesting both pathways are critical to ALS (Labarre et al., 2022).

*C. elegans* models of FUS have identified mechanisms contributing to FUS-driven ALS including FUS mislocalization, autophagic disruption, and protein synthesis dependent synaptic dysfunction. In addition, work using *C. elegans* models have identified a number of protective genes and compounds.

### Other ALS/FTLD-TDP-associated genes and risk factors

While mutations in *TARDBP, C9orf72*, *SOD1*, and *FUS* are the most common causes of fALS, there are more than 45 fALS or disease modifying risk factor genes (Smukowski et al., 2022). So far, 14 have been explored in *C. elegans* models. Briefly, evidence from *C. elegans* suggest mutations in *CHCHD10, ALS2, DCTN1*, *ELP3, TUBA4A,* and *CAV1* are loss of function mutations, which could be rescued by supplementation with the wild-type protein (Parker et al., 2007; Pan et al., 2011; Nawa et al., 2012; Woo et al., 2017; Kawamura and Maruyama, 2019; Soh et al., 2020; Ryan et al., 2021; Ryan and Hart, 2021). On the other hand, mutations in *UBQLN2*, *ATXN3,* and *TIA1* indicate a gain of function mechanism in *C. elegans* models of ALS (Fardghassemi et al., 2017; Andrusiak et al., 2019; Saxton and Kraemer, 2021). Evidence exists in *C. elegans* that both gain and loss of function mechanisms could be at play in neurodegeneration by fALS gene *HnRNPA2B1*, *KIF5A,* and *VABP* (Han et al., 2013; Zhang et al., 2017). Finally, the role of *GRN* and *RAB38* mutations in FTLD have been studied in *C. elegans* models (Grill et al., 2007; Salazar et al., 2015; Doyle et al., 2021).

Mutations in the *CHCHD10* gene cause fALS in several families. *CHCHD10* encodes a mitochondrial intermembrane protein involved in mitochondria organization (Bannwarth et al., 2014; Johnson et al., 2014; Zhang et al., 2015). Loss of *C. elegans CHCHD10* homolog, *har-1(gk3124)*, leads to motor deficits, a reduction in lifespan, and reduced mitochondrial health. The general expression of human CHCHD10, but not mutant CHCHD10(R15L) or CHCHD10(S59L), under the *eef-1A.1* promoter rescues these defects, suggesting CHCHD10 fALS mutations are loss of function mutations (Table 6) (Woo et al., 2017).

**Table 6:** *C. elegans* models of other ALS/FTLD-TDP-associated genes and risk factors.

Pathology	Approach	Transgene	ALS phenotypes	Publication	Follow-up
hnRNPA2	Deletion	*hrpa-1(tm781)*	Stress-induced glutamatergic neuron degeneration	Ryan et al. (2021)	Ryan and Hart (2021)
hnRNPA2	Mutant expression	P*_mec-4_*:: HRPA-1HsLC(D290V)::mScarlet	Stress-induced glutamatergic neuron degeneration	Ryan et al. (2021)	
hnRNPA2	Wildtype expression	P*_mec-4_*:: HRPA-1HsLC(WT)::mScarlet	N/A	Ryan et al. (2021)	
BTBD10	Deletion	*btbd-10(tm3335)*	Motor deficits, touch-receptor and GABAergic motor neuron degeneration	Nawa et al. (2012)	
BTBD10	Deletion	*btbd-10(tm3607)*	Motor deficits, touch-receptor and GABAergic motor neuron degeneration	Nawa et al. (2012)	
DCTN1	Knockdown	P*_acr2_*::shRNA(*dnc-1* RNAi)::GFP	Motor deficits, axonal degeneration, defects in axonal transport, abnormal accumulation of autophagosomes	Ikenaka et al. (2013)	Ikenaka et al. (2019)
CHCHD10	Deletion	*har-1(gk3124)*	Motor deficits, lifespan reduction	Woo et al. (2017)	
CHCHD10	Wildtype expression	P*_eef-1A.1_*::CHCHD10::BFP	N/A	Woo et al. (2017)	
CHCHD10	Mutant expression	P*_eef-1A.1_*::CHCHD10(R15L)::BFP	N/A	Woo et al. (2017)	
CHCHD10	Mutant expression	P*_eef-1A.1_*::CHCHD10(S59L)::BFP	N/A	Woo et al. (2017)	
ELP3	Deletion	*elpc-3(ok2452)*	Motor deficits, neuron dysfunction	Chen et al. (2009)	Kawamura and Maruyama (2019)
TUBA4A	Deletion	*mec-12(e1605)*	Touch-receptor neuron dysfunction	Pan et al. (2011)	
CAV1	Deletion	*cav-1(ok2089)*	Neuron dysfunction	Parker et al. (2007)	
Ubiquilin-2	Wildtype expression	P*_rgef-1_*::UBQLN2(WT)	Motor deficits, GABAergic motor neuron degeneration	Saxton and Kraemer (2021)	
Ubiquilin-2	Mutant expression	P*_rgef-1_*::UBQLN2(P506T)	Motor deficits, GABAergic motor neuron degeneration	Saxton and Kraemer (2021)	
Ubiquilin-2	Mutant expression	P*_rgef-1_*::UBQLN2(P497H)	Motor deficits, GABAergic motor neuron degeneration	Saxton and Kraemer (2021)	
Ubiquilin-2	Deletion	*ubql-1(tm1574)*	N/A	Jablonski et al. (2015)	Saxton and Kraemer (2021), Osborne et al. (2021)
ATXN3	Wildtype expression	P*_unc-47_*::ATXN3	Motor deficits, reduced lifespan	Fardghassemi et al. (2017)	
TIA1	Overexpression	P*_mec-4_*::TIAR-2	Axonal defects	Andrusiak et al. (2019)	
KIF5A	Deletion	*unc-116(e2310)*	Motor deficits, morphological defects in cholinergic motor neurons	Soh et al. (2020)	
KIF5A	Wildtype expression	P*_mec-7_*::KIF5A	NA	Nakano et al. (2022)	
KIF5A	Mutant expression	P*_mec-7_*::KIF5A(∆exon27)	Touch-receptor neuron degeneration	Nakano et al. (2022)	
VABP	Wildtype expression	P*_unc-4_*::VAPB(WT)	Motor deficits, cholinergic motor neuron degeneration	Zhang et al. (2017)	
VABP	Mutant expression	P*_unc-4_*::VAPB(P56S)	Motor deficits, cholinergic motor neuron degeneration	Zhang et al. (2017)	
Progranulin	Deletion	*pgrn-1*(tm985)	Exacerbated motor deficits in TDP-43 model	Salazar et al. (2015)	Doyle et al. (2021), Butler et al. (2019), Wang et al. (2023a)
Progranulin	Deletion	*pgrn-1(gk123284)*	Progressive motor deficits, lifespan reduction	Doyle et al. (2021)	
Progranulin	Wildtype expression	P*_pgrn-_1::*Granulin(TMdomain 1):: mCherry	N/A	Salazar et al. (2015)	
Progranulin	Wildtype expression	P*_pgrn-_1::*Granulin(TMdomain 2):: mCherry	Exacerbated motor deficits, lifespan reduction in TDP-43 model	Salazar et al. (2015)	
Progranulin	Wildtype expression	P*_pgrn-_1::*Granulin(TMdomain 3):: mCherry	Exacerbated motor deficits, lifespan reduction in TDP-43 model	Salazar et al. (2015)	
Progranulin	Overexpression	P*_pgrn-_1::pgrn-1*::mCherry	NA	Butler et al. (2019)	Wang et al. (2023a)
Progranulin	Wildtype expression	P*_pgrn-_1::*granulin1::FLAG:: mCherry	NA	Butler et al. (2019)	Wang et al. (2023a)
Progranulin	Wildtype expression	P*_pgrn-_1::*granulin2::FLAG:: mCherry	NA	Butler et al. (2019)	Wang et al. (2023a)
Progranulin	Wildtype expression	P*_pgrn-_1::*granulin3::FLAG:: mCherry	Exacerbated TDP-43 aggregation in TDP-43 model	Butler et al. (2019)	Wang et al. (2023a)
RAB38	Deletion	*glo-1(zu437)*	Axonal defects	Grill et al. (2007)	
RAB38	Deletion	*glo-1(zu391)*	Axonal defects	Grill et al. (2007)	

The *ALS2* gene encodes the Rho guanine nucleotide exchange factor Alsin, which regulates the GTPase Rab5, endosome trafficking, and neurite outgrowth. Mutations in *ALS2* cause a juvenile onset form of fALS (Yang et al., 2001; Mintchev et al., 2009; Sheerin et al., 2014). While *C. elegans* do not have a homolog of the *ALS2* gene, they do have a homolog of *BTBD10,* an Akt kinase activator. Overexpression of BTBD10, which is reduced in ALS motor neurons, can overcome *ALS2* fALS mutations by preventing Akt3 dephosphorylation (Table 6) (Nawa et al., 2008, 2012; Kanekura et al., 2004, 2005). In *C. elegans*, loss of function mutations in BTBD10 homolog *btbd-10(tm3335)* and *btbd-10(tm3607)* result in motor deficits and degeneration of touch-receptor and GABAergic motor neurons, creating a possible unique model of juvenile ALS (Table 6) (Nawa et al., 2012).

Mutations in the gene *DCTN1* can cause fALS. The *DCTN1* protein, dynactin-1, activates the microtubule-binding motor protein dynein (Gill et al., 1991; Puls et al., 2003; Munch et al., 2004). In *C. elegans*, the knock-down of *DCTN1* homolog *dnc-1* in motor neurons by the expression of an RNAi construct under the *acr-2* promoter results in motor deficits, axonal degeneration, defects in axonal transport, and the abnormal accumulation of autophagosomes (Table 6) (Ikenaka et al., 2013). A drug screen in this model identified 12 neuroprotective compounds, two of which (ALS-approved drugs riluzole and nifedipine) also rescue motor deficits in a *C. elegans* model of TDP-43 toxicity (Ikenaka et al., 2019).

Mutations in the elongator acetyl transferase complex subunit 3 (ELP3) are associated with sALS in several genome wide associations screens and decrease survival in cases of C9orf72 fALS (Simpson et al., 2009; Kwee et al., 2012). Part of the elongation complex for RNA polymerase II, ELP3 is known to acetylate alpha-tubulin and modify tRNA uridines, regulating mTORC2 activation and other biological pathways (Creppe et al., 2009; Chen et al., 2022). Deletion of the *C. elegans* homolog of ELP3, *elpc-3(ok2452)*, results in progressive motor deficits (Kawamura and Maruyama, 2019). A second deletion allele, *elpc-3(tm3120)*, also disrupts experience-dependent learning, neuropeptide signaling, and tRNA modification, suggesting ELP3 loss of function contributes to ALS (Chen et al., 2009). In a cell model of SOD1 ALS, ELP3 depletion increased SOD1 aggregation (Bento-Abreu et al., 2018).

Mutations in the tubulin gene tubulin alpha 4a (*TUBA4A*) have been identified as likely pathogenic in several rare cases of fALS and FTLD-TDP (Smith et al., 2014; Mol et al., 2021). TUBA4A is a tubulin alpha chain, one of the basic building blocks of microtubules and the cytoskeleton (Ganne et al., 2023). A mutation in the *C. elegans TUBA4A* homolog, *mec-12(e1605)*, leads to progressive neuronal dysfunction in touch receptor neurons but does not alter lifespan (Pan et al., 2011). Structural and biological studies suggest TUBA4A mutations have both a loss of function component, disrupting microtubule polymerization, as well as a gain of function component, where mutant TUBA4A aggregates and disrupts microtubule dynamics in fALS (Smith et al., 2014; Ganne et al., 2023).

Mutations in the enhancer region of the *CAV1* gene are a risk factor for the development of ALS. These mutations decrease the expression of *CAV1* in patient-derived neurons, which encodes for a critical plasma membrane structural protein named caveolin-1 (Cooper-Knock et al., 2020). In *C. elegans*, the deletion of *CAV1* homolog *cav-1(ok2089)* results in increased sensitivity to levamisole, suggesting defects in neuromuscular junction or muscle function (Table 6). Depletion of *cav-1* by RNAi also worsens a temperature-sensitive motor deficit in *dyn-1(ky51)* mutants. Furthermore, mutations in *cav-1* alter the distribution of the *dyn-1* protein dynamin, a regulator of vesicle trafficking (Parker et al., 2007). Together these results suggest *CAV1* acts in ALS by disrupting motor neuron function at the synapse.

Mutations in the *Ubiquilin-2 (UBQLN2)* gene are responsible for fALS cases in several families (Deng et al., 2011; Williams et al., 2012; Gellera et al., 2013; Fahed et al., 2014). UBQLN2 shuttles ubiquitinated proteins to the proteasome for degradation. Evidence suggests fALS mutations in UBQLN2 impair autophagy, mitochondrial function, and the delivery of UBQLN2 substrates to the proteasome (Lin et al., 2022). In *C. elegans*, the neuronal expression of wild-type human UBQLN2 and mutant UBQLN2(P506T) and UBQLN2(P497H) under the *rgef-1* promoter results in motor deficits and degeneration of GABAergic motor neurons. *C. elegans tdp-1* is not required for these phenotypes (Table 6). Co-expression of wild-type and mutant UBQLN2 with wild-type human TDP-43 exacerbates motor deficits, neurodegeneration, and increases the accumulation of TDP-43 and UBQLN2 (Saxton and Kraemer, 2021). A loss of function mutation in the UBQLN2 homolog *ubql-1(tm1574)* has no effect on the motor performance of a *C. elegans* model expressing mutant TDP-43 or on neurodegeneration in a *C. elegans* model expressing SOD-1(G85R) (Jablonski et al., 2015; Osborne et al., 2021).

Several genome-wide association studies in ALS patients have identified mutations in *ATXN3* that increase the risk of developing ALS. *ATXN3* encodes the protein Ataxin-3, an important player in protein quality control during stress. Expansions in a CAG repeat region in *ATXN3* cause Machado-Joseph disease, another progressive motor disorder. ALS-associated risk mutations in ATXN3 are also thought to impact this region (Nakamura et al., 2020; Li et al., 2021; Humphrey et al., 2023). The expression of human *ATXN3* with 10 CAG repeats, below the threshold to cause Machado-Joseph disease, in *C. elegans* GABAergic neurons under the *unc-47* promoter results in motor deficits and reduced lifespan, but not neurodegeneration (Table 6) (Fardghassemi et al., 2017). The *C. elegans* homolog *atx-3* regulates autophagy, and when deleted, enhances the stress response to heat and extends lifespan of *cdc-48.1/VCP* mutants (Kuhlbrodt et al., 2011; Rodrigues et al., 2011; Herzog et al., 2020).

Mutations in *TIA1* have been identified in several cases of ALS (Hirsch-Reinshagen et al., 2017; Mackenzie et al., 2017). *TIA1* encodes the protein TIA1 which regulates various aspects of RNA metabolism and stress granules dynamics (Rayman and Kandel, 2017), and ALS-associated mutations may enhance TIA1 phase separation and aggregation in disease (Mackenzie et al., 2017; Sekiyama et al., 2022). In *C. elegans*, overexpression of the *TIA1* homolog, *tiar-2*, in mechanosensory neurons under the *mec-4* promoter inhibits axon regeneration following injury. TIAR-2 phase separates into dynamic granules in *C. elegans* neurons (Table 6). Granule formation is required for TIAR-2’s suppression of axon regeneration and is regulated by phosphorylation of TIAR-2’s prion-like domain (Andrusiak et al., 2019).

Mutations in the *HnRNPA2B1* and *HnRNPA1* genes are responsible for fALS in several families (Kim et al., 2013). The *HnRNPA2B1* and *HnRNPA1* genes express several protein isoforms, including HNRNPA2, HNRNPA1, and HNRNPA3, which control mRNA splicing, trafficking, stability, and translation (Hutchison et al., 2002; Fahling et al., 2006; Kosturko et al., 2006; Clower et al., 2010). A loss of function mutation in the *C. elegans HnRNPA2B1* and *HnRNPA1* homolog gene *hrpa-1(tm781)* results in glutamatergic neuron degeneration (Ryan et al., 2021; Ryan and Hart, 2021). The expression of a chimeric protein of the *C. elegans* HRPA-1 and the human low complexity domain of mutant hnRNPA2(D290V) in glutamatergic neurons under the *mec-4* promoter results in stress-induced neurodegeneration of glutamatergic neurons (Table 6). Neurodegeneration is rescued by loss of *tdp-1*. The expression of a chimera of HRPA-1 and the wild-type human low complexity domain does not result in neurodegeneration. Post translational modification of hnRNPA2 can influence its phase separation and aggregation, impacting ALS progression (reviewed in Farina et al., 2021). In *C. elegans*, the co-expression of a constitutively active Fyn kinase, which phosphorylates hnRNPA2, and the chimera HRPA-1 hnRNPA2(D290V) protein reduces stress-induced glutamatergic neuron neurodegeneration (Ryan et al., 2021). This suggests hnRNPA2 phosphorylation may be a therapeutic target in fALS.

Mutations in the C-terminal cargo-binding domain of the protein KIF5A cause fALS in several families (Brenner et al., 2018; Nicolas et al., 2018; Saez-Atienzar et al., 2020). KIF5A, or kinesin-1, is a microtubule motor protein involved in intracellular trafficking and is necessary for neuronal development and function (Wang and Brown, 2010; Nakajima et al., 2012). In *C. elegans*, loss of KIF5A homolog *unc-116(e2310)* results in progressive motor deficits and morphological defects in cholinergic motor neurons, further suggesting defects in axonal transport are key in ALS (Table 6) (Soh et al., 2020). The expression of a human fALS KIF5A splicing variant, KIF5A(∆exon27), in *C. elegans* touch-receptor neurons under the *mec-7* promoter results in morphological defects and degeneration of touch receptor neurons. *C. elegans* expressing wildtype KIF5A do not show any defects, indicating KIF5A(∆exon27) has a toxic gain of function (Table 6) (Nakano et al., 2022).

A point mutation in the *VAPB* gene causes a late-onset form of fALS in several families (Nishimura et al., 2004; Millecamps et al., 2010). VAPB is found in the ER-membrane and regulates Golgi transport, neurotransmitter release, and calcium homeostasis (Skehel et al., 1995; Soussan et al., 1999; Amarilio et al., 2005; De Vos et al., 2012; Morotz et al., 2012). RNAi knock-down of the *C. elegans* homolog *vpr-1* results in stress-inducible motor deficits and stress-inducible cholinergic motor neuron degeneration, suggesting VAPB loss of function may drive fALS (Table 6). PIK-93, a synthetic PI4K inhibitor, partially prevents these stress-induced motor deficits and motor neuron degeneration when delivered at a concentration of 250 nM via liquid culture. The expression of both human wild-type VAPB and mutant VAPB(P56S) in *C. elegans* cholinergic motor neurons under the *unc-4* promoter also results in age-dependent motor deficits and progressive degeneration of cholinergic motor neurons (Table 6) (Zhang et al., 2017).

Mutations in the progranulin gene, whose protein regulates a diverse array of biological functions from cell growth and survival to repair and inflammation, cause frontotemporal lobar degeneration (FTLD) in several families (Baker et al., 2006; Cruts et al., 2006; Moore et al., 2020). Progranulin is cleaved into individual granulin peptides, a process that is disrupted in FTLD. A reduction in progranulin protein levels overall is also thought to drive disease by disrupting endolysosomes, lysosomal homeostasis, inflammation, and other pathways (Ward and Miller, 2011; Mohan et al., 2021; Amin et al., 2022). Deletion of the *C. elegans* progranulin homolog, *pgrn-1*, results in progressive motor deficits, a reduction in lifespan, and lysosomal and autophagic defects, indicating progranulin loss of function may contribute to disease (Table 6). A deletion of *sphk-1(ok1097)*, whose human homolog SPHK1 regulates sphingolipid metabolism, restores autophagosome and autolysosome numbers in *pgrn-1* mutants. RNAi knockdown of *cgt-3/UGCG* and *asah-1/ASAH1*, also involved in sphingolipid metabolism, rescue *prgn-1* mutant motor deficits. A screen of molecular compounds in this model uncovered two drugs, Rivastigmine and Rottlerin, which rescue *pgrn-1* mutant motor and autophagy deficits and had a prolonged positive effect in a cell culture model (Doyle et al., 2021). In another *C. elegans* model expressing human TDP-43, heterozygous deletion of *pgrn-1(tm985)* worsens motor deficits. Additionally, the selective expression of progranulin cleavage products, the granulin peptides 2 and 3, but not 1, under the *pgrn-1* promoter, exacerbate motor deficits, reduce lifespan, and increase TDP-43 accumulation modeling possible interactions in FTLD-TDP (Table 6) (Salazar et al., 2015). Further studies of granulin peptides found they localize to endolysosomes, disrupt lysosomal morphology and lysosomal protease activity, and their expression inhibits ER stress resistance and impairs organismal fitness. Interestingly age and organismal stress drives increased accumulation of granulins (Butler et al., 2019). Granulins 1, 2, and 3 may have distinct phenotypic outcomes, with granulin 3 in particular impairing organismal fitness and preventing clearance of TDP-43 (Butler et al., 2019; Wang et al., 2023a).

In genome-wide association studies, mutations associated with *RAB38* are linked with FTLD, specifically in patients with personality and behavioral changes (Yang et al., 2017; Reus et al., 2021). These mutations are thought to increase the risk of FTLD by altering the expression of RAB38, which encodes the transmembrane protein RAB38 that regulates lysosomal trafficking (Bultema et al., 2012; Ferrari et al., 2014). In *C. elegans*, loss of function mutations in the *RAB38* homolog, *glo-1*, disrupt axon termination, likely resulting in neuron dysfunction (Table 6) (Grill et al., 2007).

## Conclusions and outlook

During the last 10 years of progress, *C. elegans* models of ALS and FTLD-TDP have been used to identify conserved biological pathways in disease, characterize the impact of disease-causing mutations, determine the role of post-translational modifications in disease progression, uncover novel suppressors of neurodegeneration, and screen drug libraries and novel chemical compounds in the search for new therapeutic treatments. These models use diverse genetic and transgenic strategies, but recapitulate aspects of human disease, including protein or RNA aggregation, motor deficits and neurodegeneration. These characteristics have allowed for the identification of conserved, translationally relevant, and potentially targetable biological pathways in disease.

Overall, *C. elegans* models of ALS and FTLD-TDP have fundamentally advanced our understanding of the biology driving neurodegeneration and are important tools for future studies. They will continue to be a valuable asset in characterizing the molecular consequences of novel disease-associated mutations in known and unknown genes. Compared to cell culture and mice, *C. elegans* have robust behavioral, neurodegenerative, and lifespan phenotypes and are cost-effective for high throughput screens. Their short generation time enables aging studies not feasible in other systems. Recognition of limitations of the model system are also critical for interpreting results. While *C. elegans* lack a conserved neuroinflammatory pathway and canonical glia, both important in ALS pathology, previous work in *C. elegans* has been able to identify a key role for the innate immune response in ALS models. Additionally, glia-like cells in *C. elegans* have been shown to regulate proteostasis and stress responses in neurons and could be studied in ALS models (Veriepe et al., 2015; Bar-Ziv et al., 2023; Wang et al., 2023b). For abnormal protein conformations and inclusions that slowly develop over time, the short lifespan of *C. elegans* may not be long enough to observe mature pathological species. However, *C. elegans* models of ALS and FTLD-TDP will continue to be leveraged to screen novel neuroprotective compounds, identify novel genetic targets for therapeutic intervention, and identify key biological pathways necessary for neurodegeneration which can be further explored in human cells, vertebrate animal models, and patient tissue. *C. elegans* models enable evaluation of the impacts of stress, environment, and chemical insults on disease progression. Robust tools for genetic manipulation will continue to drive the development of new and refined *C. elegans* systems that will continue to contribute to understanding the biology of ALS.

## Author contributions

RE: Conceptualization, Visualization, Writing – original draft, Writing – review & editing. JS: Writing – original draft, Writing – review & editing. BK: Writing – review & editing, Funding acquisition, Project administration, Resources, Supervision, Validation. NL: Funding acquisition, Project administration, Resources, Supervision, Writing – review & editing, Conceptualization, Visualization, Writing – original draft.
